# Trimethylamine N-oxide and Homocysteine in Kidney Function and Disease: Distinct Metabolic Origins, Shared Renal Determinants and Pathophysiological Pathways

**DOI:** 10.3390/ijms27177517

**Published:** 2026-08-22

**Authors:** Monica Currò, Maria Paola Bertuccio, Riccardo Ientile, Daniela Caccamo

**Affiliations:** Department of Biomedical and Dental Sciences and Morpho-Functional Imaging, University of Messina, 98125 Messina, Italy; monica.curro@unime.it (M.C.); ientiler@gmail.com (R.I.); daniela.caccamo@unime.it (D.C.)

**Keywords:** Trimethylamine N-oxide, homocysteine, kidney disease, cardiovascular risk, biomarkers

## Abstract

Trimethylamine N-oxide (TMAO) and homocysteine are key biomarkers linked to cardiovascular and chronic kidney disease (CKD). Although they originate from distinct metabolic pathways, their circulating levels are jointly shaped by renal clearance, systemic metabolic status, and nutrient availability, complicating their biological interpretation. TMAO derives from gut microbiota–dependent metabolism of dietary precursors followed by hepatic oxidation of trimethylamine, whereas homocysteine is a central intermediate of one-carbon metabolism governed by remethylation and transsulfuration. Choline and betaine provide a nutritional interface between these pathways because choline can contribute to microbial TMA production and betaine acts as a methyl donor for homocysteine remethylation via betaine–homocysteine methyltransferase (BHMT). However, this interface does not establish direct metabolic interdependence between circulating TMAO and homocysteine. Declining renal function promotes the accumulation of both TMAO and homocysteine, although their renal handling and the mechanisms underlying their increase in CKD are not identical. Experimental and clinical evidence also links both metabolites to partially overlapping downstream processes, including oxidative stress, inflammation, endothelial dysfunction, and fibrotic remodeling. These shared pathophysiological responses should be distinguished from convergence of their biosynthetic pathways. This narrative review examines how renal function, metabolic regulation, and nutritional factors influence TMAO and homocysteine biology, emphasizing renal handling, analytical variability, shared downstream mechanisms, and the limitations of isolated measurements. A shift toward context-aware, multiparametric assessment may enhance their interpretation in cardiorenal research, although neither biomarker currently replaces established measures of kidney function.

## 1. Introduction

Cardio-renal biomarkers reflecting the interplay between metabolism and kidney function represent a major challenge in clinical biochemistry. In the realm of contemporary metabolomics, TMAO and homocysteine have emerged as notable subjects of investigation, given their consistent associations with cardiovascular and chronic kidney disease [1,2,3,4,5,6]. These associations require cautious interpretation because kidney function is itself a major determinant of both circulating concentrations.

Despite their provenance from discrete biochemical pathways, these metabolites exhibit numerous clinically pertinent characteristics. Both parameters are influenced by dietary and metabolic factors, and both are strongly affected by renal function. Furthermore, both have been proposed as biomarkers of cardiovascular risk. However, the interpretation of these findings in routine laboratory practice remains challenging, given that circulating concentrations reflect the combined effects of production, metabolism, and renal clearance.

TMAO is a gut microbiota-derived metabolite generated through a metaorganismal pathway involving dietary precursors such as choline, phosphatidylcholine, betaine, and L-carnitine, followed by hepatic oxidation via flavin-containing monooxygenases 3 (FMO3) [6,7]. Recent evidence further supports its role as a link between gut dysbiosis and cardiometabolic disease, reinforcing its relevance as a systemic metabolic marker rather than a single-pathway metabolite [8]. In contrast, homocysteine is an intermediate of one-carbon metabolism, which is regulated by remethylation and transsulfuration pathways that depend on folate and B-vitamin availability [9,10].

Renal function is a significant determinant of circulating concentrations of both metabolites. TMAO is primarily cleared by the kidneys, and its levels progressively increase with declining estimated glomerular filtration rate (eGFR) and CKD severity [11,12,13,14]. A bidirectional relationship has been proposed, whereby renal dysfunction promotes TMAO accumulation, while elevated TMAO may contribute to inflammation, oxidative stress, fibrosis, and CKD progression [15,16]. However, evidence for a causal contribution in humans remains less conclusive than the evidence for accumulation and association. Similarly, hyperhomocysteinemia (hHCY) is common in CKD, but its increase reflects altered renal and extrarenal metabolism rather than simple failure to excrete filtered homocysteine [4].

At the molecular level, both TMAO and homocysteine have been implicated in signaling pathways associated with cardiovascular and renal injury. Experimental studies suggest that TMAO may activate nuclear factor-kappa B (NF-κB), NOD-like receptor protein 3 (NLRP3) inflammasome signaling, transforming growth factor-β (TGF-β)/Smad pathways, and reactive oxygen species (ROS)-dependent mechanisms, thereby promoting inflammation, fibrosis, and endothelial dysfunction [17,18,19,20].

Likewise, hHCy has been associated with oxidative stress, endothelial injury, impaired nitric oxide bioavailability, endoplasmic reticulum stress, mitochondrial dysfunction, and alterations in cellular methylation capacity through disruption of S-adenosylmethionine (SAM) and S-adenosylhomocysteine (SAH) homeostasis [21,22,23,24]. These molecular mechanisms have been implicated in vascular remodeling, inflammation, and progressive renal damage [25,26].

The overlap between these downstream responses represents a potential pathophysiological convergence. It does not imply that TMAO and homocysteine share a common biosynthetic pathway or that one directly regulates the circulating concentration of the other.

Despite extensive investigation, the interpretation of both biomarkers remains controversial. Elevated circulating TMAO levels (≥5 µmol/L) have been consistently associated with adverse cardiovascular and renal outcomes [12], although the relative contributions of increased production versus reduced renal clearance remain incompletely understood [13]. Similarly, hHCy has been identified as a feature of CKD and cardiovascular disease, yet the causal role and independent clinical utility of this condition remain the subject of debate.

Choline and betaine provide an additional biochemical interface between TMAO-generating pathways and one-carbon metabolism. Choline can serve as a substrate for microbial TMA formation or be oxidized by the host to betaine; betaine then supports BHMT-dependent remethylation of homocysteine [27,28]. This shared dependence on selected nutrients should not be interpreted as evidence of direct metabolic competition between TMAO production and homocysteine clearance.

The present review aims to provide an integrated clinical biochemistry framework for the interpretation of both TMAO and homocysteine concentrations, with particular emphasis on their distinct metabolic origins, renal handling, nutritional determinants, and partially overlapping downstream effects. Specifically, this review discusses the analytical, pre-analytical, and physiological determinants influencing circulating levels of these biomarkers, and addresses the challenge of distinguishing increased production, altered metabolism, and reduced renal clearance in clinical and research settings.

## 2. Comparative Metabolism of TMAO and Homocysteine

TMAO and homocysteine originate from distinct metabolic pathways, although selected nutrients and physiological determinants influence both systems. Understanding their biochemical origins is necessary for interpreting their circulating concentrations within the context of clinical biochemistry in order to accurately interpret them.

TMAO is produced through a metagenomic pathway that involves both the intestinal microbiota and hepatic metabolism. Dietary precursors such as choline, phosphatidylcholine, betaine, and L-carnitine are metabolized by gut bacteria into TMA, which is subsequently absorbed and oxidized in the liver by FMO3 to form TMAO [6,7]. This pathway explains the dependence of TMAO production on dietary composition and gut microbiota structure, both of which contribute to substantial interindividual variability. In human intervention studies, broad-spectrum antibiotics markedly suppressed TMAO generation after a phosphatidylcholine challenge, with recovery after antibiotic withdrawal, supporting a direct contribution of the intestinal microbiota [6].

The kidney is a critical determinant of TMAO homeostasis. Once formed, TMAO is primarily eliminated through renal excretion; therefore, declining kidney function leads to progressive TMAO accumulation in circulation [6,11]. The close dependence on renal clearance thus links TMAO metabolism to the gut–kidney axis, a bidirectional network in which gut-derived metabolites influence renal function, while kidney dysfunction modifies the intestinal microenvironment and promotes the retention of microbial metabolites [29,30,31]. In this context, TMAO may act not only as a marker of gut microbial metabolism but also as a retention solute whose circulating levels are strongly shaped by renal function [13].

Homocysteine is an intermediate of one-carbon metabolism arising from the demethylation of methionine. Within the methionine cycle, methionine is converted to SAM, followed by its conversion to SAH, and subsequently to homocysteine [32]. Homocysteine can be remethylated to methionine through folate- and vitamin B12-dependent pathways or be irreversibly catabolized through the vitamin B6-dependent transsulfuration pathway [32,33]. Under normal dietary conditions, the remethylation pathway converts approximately 40% of homocysteine body pool to maintain continuous methionine and SAM supplies for protein synthesis and methylation. An additional remethylation pathway uses betaine as a methyl donor through BHMT, particularly in the liver and kidney [27]. This regulation links circulating homocysteine concentrations to nutritional status, intracellular methylation balance, genetic determinants, and renal function.

Despite their different origins, TMAO and homocysteine have several determinants in common, including nutritional status, hepatic metabolism, and kidney function. Choline and betaine provide a biochemical interface: these compounds can contribute to microbial TMA production, while betaine also functions as a methyl donor for BHMT-dependent remethylation of homocysteine [27,28]. This interface reflects alternative uses of selected nutrients but does not demonstrate that increased TMAO production causes impaired homocysteine metabolism.

The liver also contributes to both systems. Hepatic metabolism governs both the oxidation of TMA to TMAO and key reactions within one-carbon metabolism. Experimental CKD has been associated with increased hepatic FMO3 expression and TMAO formation, suggesting that CKD-related TMAO elevation may involve altered production in addition to reduced elimination; confirmation in humans is still required [34].

A fundamental distinction concerns the relative contribution of exogenous and endogenous determinants. TMAO is sensitive to recent dietary intake and gut microbiota composition, resulting in short-term variability [6,35]. Homocysteine is regulated by endogenous metabolic pathways but is also influenced by B-vitamin status and renal function [36]. It would nevertheless be an oversimplification to classify TMAO exclusively as a short-term biomarker and homocysteine as a long-term biomarker, because both exhibit acute and chronic biological determinants.

**Figure 1:**
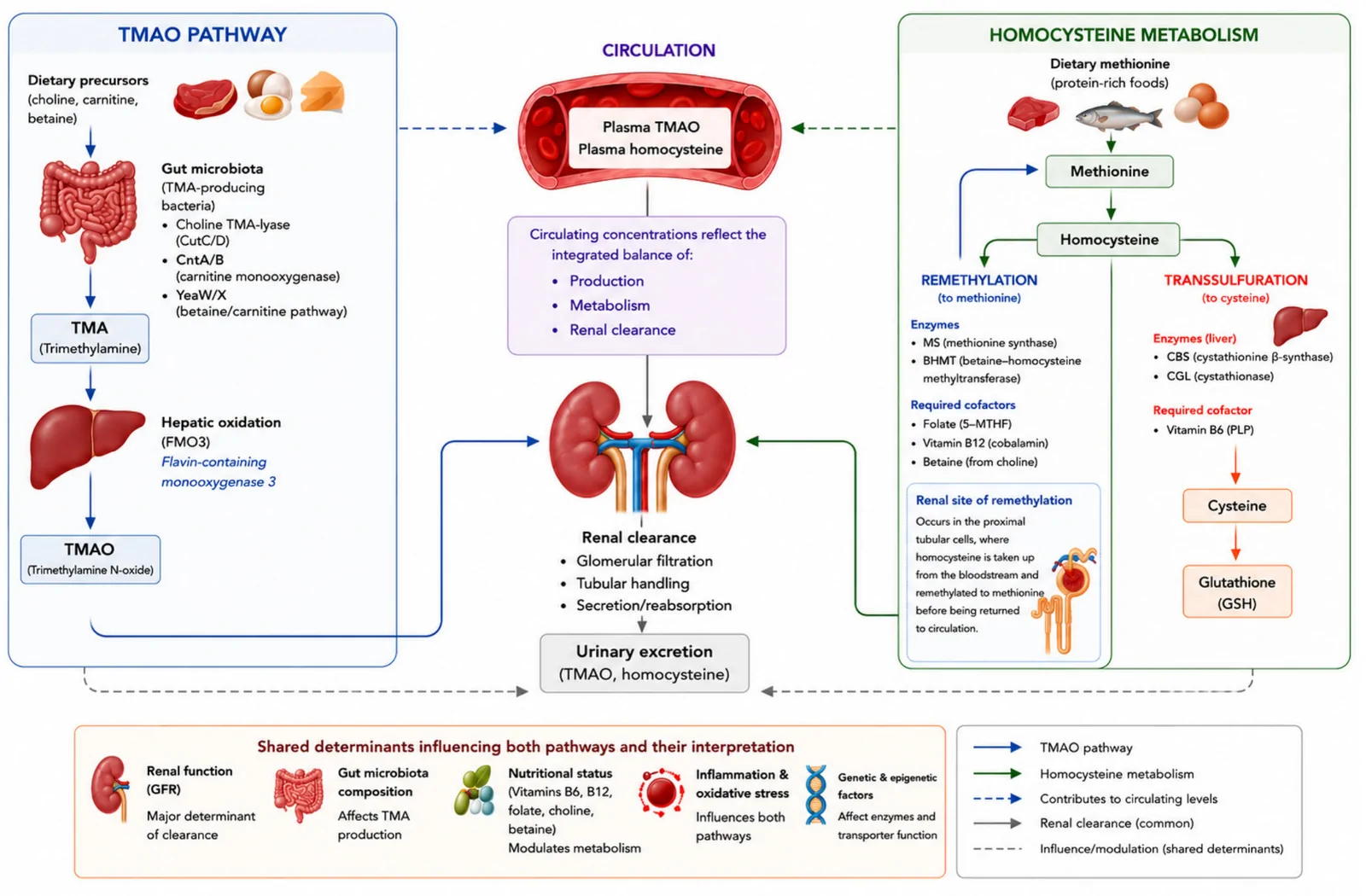
Integrated but distinct metabolic pathways of TMAO and homocysteine and their relationships with kidney function. TMAO is generated through dietary precursor availability, microbial TMA formation, and hepatic FMO3-dependent oxidation, whereas homocysteine arises from methionine and one-carbon metabolism. Choline and betaine provide a nutritional interface, but current evidence does not demonstrate direct metabolic interdependence between circulating TMAO and homocysteine. Renal function, nutritional status, and metabolic factors contribute to interindividual variability and interpretative complexity.

Consequently, elevated TMAO levels may be indicative of recent dietary exposure, gut microbiota-dependent production, or reduced renal clearance. In contrast, elevated homocysteine levels are more frequently associated with altered one-carbon metabolism, nutritional deficiency, impaired renal handling, or a combination of these factors. In both cases, circulating concentrations are the integrated outcome of production, metabolism, and elimination, which reinforces the need for an interpretative approach that accounts for kidney function and metabolic context (Figure 1).

## 3. Renal Handling as a Determinant of Circulating TMAO and Homocysteine Accumulation

Renal function is a major determinant of circulating concentrations of both TMAO and homocysteine, thereby influencing their biological and clinical interpretation. However, the renal handling of the two metabolites is not identical, and their concurrent increase in CKD should not be interpreted as evidence of a shared clearance mechanism.

TMAO is primarily eliminated through the kidneys, and circulating concentrations increase progressively as GFR declines [12,13]. Clinical clearance data indicate that glomerular filtration is quantitatively predominant; TMAO clearance was close to measured GFR, with a mean fractional excretion of approximately 105% [37]. In advanced CKD, reduced filtration results in marked accumulation, with plasma TMAO concentrations often several-fold higher than in individuals with preserved renal function [11,13].

Experimental studies nevertheless identify a potential tubular transport pathway. Organic cation transporter 2 (OCT2), located mainly at the basolateral membrane of proximal tubular cells, can mediate TMAO uptake from blood, whereas multidrug and toxin extrusion protein 1 (MATE1) can contribute to apical efflux into the tubular lumen [38,39,40]. These findings demonstrate transporter capacity, but human clearance studies suggest that net tubular secretion is modest under the conditions examined. Species differences are also relevant, because OCT-dependent secretion appears more prominent in mice than in humans.

Beyond reduced renal elimination, experimental evidence suggests that CKD may increase hepatic FMO3 expression and activity, thereby enhancing TMAO formation. This observation indicates that CKD-related TMAO elevation may not reflect passive retention alone, although the evidence currently derives from animal models [34].

TMAO has also been proposed to actively participate in renal injury. Experimental studies associate TMAO with ROS-dependent NLRP3 inflammasome activation, pro-inflammatory cytokine release, and endothelial dysfunction [17,19]. In vascular endothelial and smooth muscle cells, TMAO has also been shown to activate MAPK and NF-κB signaling and to induce inflammatory gene expression [41]. These findings support biological plausibility, but do not establish that circulating TMAO is an independent causal driver of CKD progression in humans.

Homocysteine exhibits a more complex relationship with kidney function. Because most circulating homocysteine is protein-bound and urinary excretion is limited, CKD-associated hHCy cannot be explained simply by reduced filtration of free homocysteine. The kidney participates in homocysteine metabolism, but an arterio–venous study found no significant net renal extraction in fasting humans with preserved renal function [42]. Consequently, hHCy in CKD probably reflects altered renal and extrarenal metabolism, reduced metabolic clearance, tissue release, nutritional status, and other changes associated with the uremic state [4].

In addition to its accumulation in CKD, homocysteine may actively contribute to renal injury through several molecular mechanisms. Elevated homocysteine promotes oxidative stress by increasing ROS generation and impairing antioxidant defenses, thereby contributing to endothelial dysfunction and reduced nitric oxide bioavailability [24,43]. Homocysteine has also been shown to induce endoplasmic reticulum stress, mitochondrial dysfunction, and activation of apoptotic pathways involving Bax/Bcl-2 imbalance and caspase signaling [44]. hHCy may also alter cellular methylation capacity through accumulation of SAH, a potent inhibitor of methyltransferases. This phenomenon can lead to epigenetic dysregulation, altered gene expression, and activation of pro-inflammatory and pro-fibrotic pathways [45]. Several studies further suggest activation of TGF-β signaling, extracellular matrix deposition, and glomerulosclerosis, thereby contributing to progressive renal fibrosis [46,47]. As with TMAO, much of this evidence is experimental, and causal relevance at circulating concentrations observed in human CKD remains incompletely defined. Collectively, these observations indicate that homocysteine may act not only as a marker of impaired renal function but also as a potential mediator of oxidative, inflammatory, and fibrotic processes involved in CKD progression.

The distinction of underlying mechanism between both metabolites in CKD is clinically relevant because similar increases in circulating concentrations may arise from different pathophysiological mechanisms. In CKD, both TMAO and homocysteine exhibit features of retention solutes [4,13,37]. However, their designation as established uremic toxins requires caution, because evidence of accumulation in humans is stronger than evidence that either metabolite independently causes uremic toxicity. According to the conventional EUTox framework, accumulation due to reduced kidney function identifies a uremic retention solute, whereas classification as a uremic toxin additionally requires evidence of biological or clinical toxicity [48,49].

Elevated circulating concentrations may therefore reflect reduced renal function rather than increased metabolic production alone. This introduces a risk of overestimating cardiovascular or metabolic risk when the biomarkers are interpreted without adequate consideration of eGFR. Neither analyte should currently be presented as a specific or superior indicator of early renal injury compared with established measures such as creatinine, cystatin C, albuminuria, and eGFR [11,49].

The available evidence also differs substantially in design and inferential strength. Cell and animal studies support mechanisms through which TMAO and homocysteine could contribute to oxidative, inflammatory, endothelial, and fibrotic injury, but they frequently use exposures or experimental conditions that cannot be directly extrapolated to human CKD. Most clinical evidence is observational and is therefore vulnerable to residual confounding and reverse causation. Experimental studies suggest that increased TMAO exposure may promote tubulointerstitial fibrosis and renal dysfunction, although these findings derive primarily from animal models [50]. Prospective cohort data showing an association between higher TMAO concentrations and incident CKD or faster eGFR decline support a temporal association but do not establish causality or demonstrate that lowering TMAO would prevent CKD [51].

Changes after renal replacement therapy further illustrate the importance of renal function. Although TMAO is efficiently removed during individual hemodialysis or hemodiafiltration sessions, marked predialysis accumulation may persist because intermittent renal replacement therapy does not reproduce the continuous high clearance provided by normally functioning kidneys [37,52]. Restoration of kidney function after renal transplantation substantially reduces circulating TMAO concentrations, although the extent of normalization varies among studies [11,53]. These observations strongly support renal function as a determinant of TMAO exposure but do not by themselves resolve whether chronic TMAO accumulation contributes independently to renal or cardiovascular injury.

For homocysteine, the discrepancy between observational and interventional evidence is particularly informative. Although higher homocysteine concentrations are associated with CKD severity and cardiovascular risk in observational studies, associations with mortality may be markedly attenuated after adjustment for GFR [54]. Moreover, a large randomized trial and a meta-analysis of randomized trials showed that folic acid and B-vitamin treatment lowered homocysteine without reducing mortality or major vascular outcomes in patients with advanced CKD or end-stage kidney disease [55,56]. This weakens a simple causal interpretation and suggests that homocysteine may partly reflect renal dysfunction and associated metabolic disturbances rather than acting as an isolated, readily modifiable driver of risk.

## 4. Integrating Production, Metabolism, and Renal Clearance in Biomarker Interpretation

**Figure 2:**
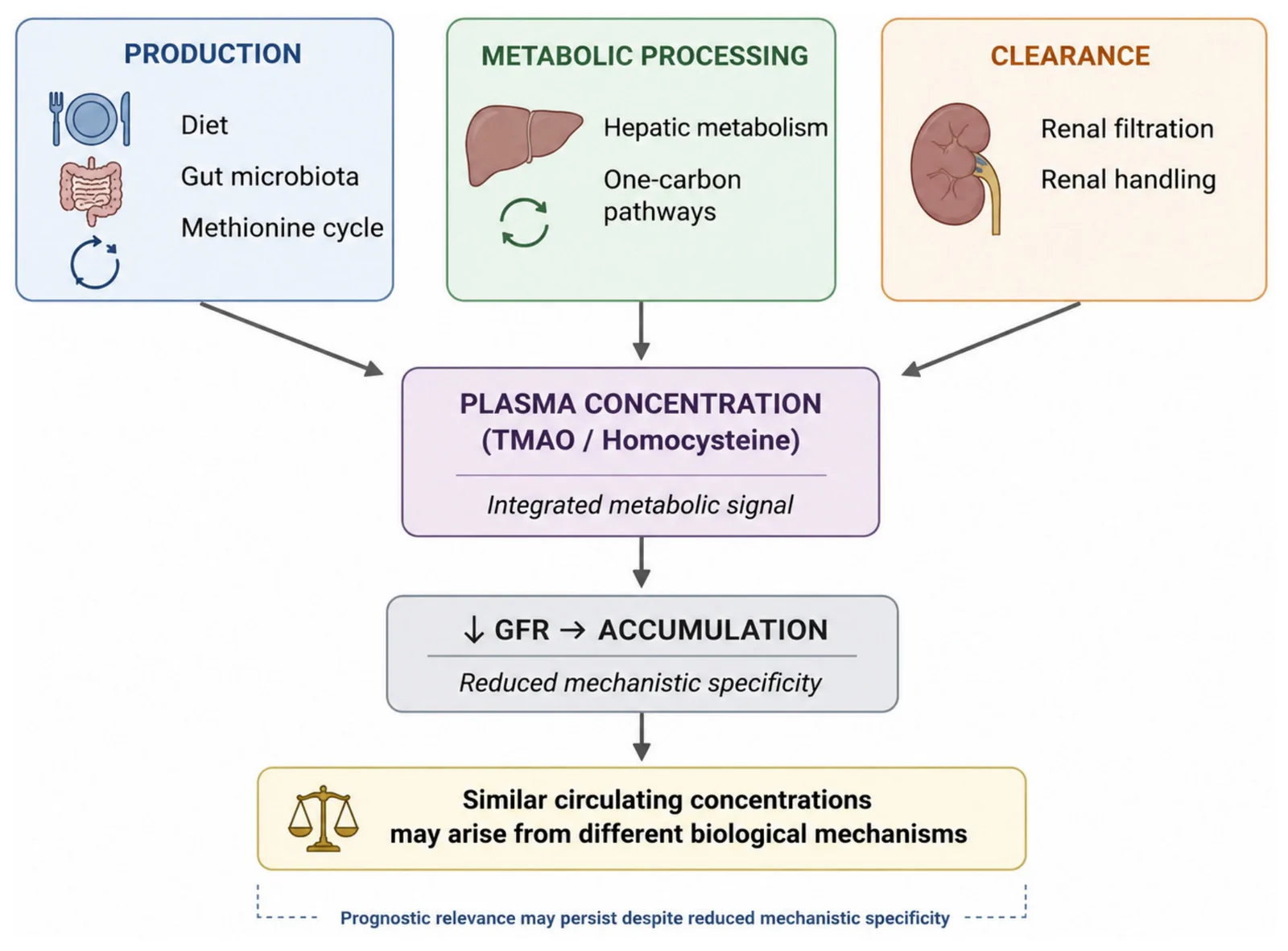
Three-component model for the interpretation of TMAO and homocysteine. Circulating concentrations reflect production, metabolic processing, and renal clearance. Declining kidney function increases the contribution of retention and reduces the specificity of a measured concentration for its upstream metabolic pathway. Prognostic information, where present, must be evaluated after appropriate adjustment for renal function and other relevant covariates.

A major challenge in interpreting circulating TMAO and homocysteine is that single plasma measurements are unable to distinguish between increased production, altered metabolism, and reduced renal clearance [4]. This is a limitation of biological interpretation, not of analytical detection. Consequently, circulating concentrations are indicative of the integrated output of multiple physiological processes rather than a single biological pathway, creating substantial interpretative ambiguity, particularly in individuals with impaired renal function [13,49]. This complexity can be conceptualised through a “three-component model”, in which circulating concentrations are determined by the combined contributions of: production, metabolism, and renal clearance (Figure 2) [4].

Within this framework, similar plasma concentrations may arise from fundamentally different biological conditions. In the case of TMAO, elevated concentrations in individuals with preserved GFR may reflect dietary exposure and gut microbiota-dependent production, whereas in renal dysfunction reduced clearance may contribute substantially [6,13]. A similar, albeit more metabolically complex, paradigm applies to homocysteine, the circulating concentrations of which may reflect disturbances in one-carbon metabolism, vitamin deficiencies, altered tissue metabolism, impaired renal handling, or combinations of these factors [4].

Diet and renal function can influence more than one component of this model, but available evidence does not justify describing their interaction as metabolic amplification or as a coordinated TMAO–homocysteine axis. Elevated concentrations may be misinterpreted as evidence of increased cardiovascular risk or metabolic dysregulation if renal function and nutritional status are not considered. Conversely, altered production may be overlooked if every increase is attributed to impaired clearance.

Interpretation should therefore adopt an integrated, context-aware approach. eGFR should be considered together with nutritional and metabolic status, including vitamin B12 and folate. Where justified by the research question, complementary metabolites such as choline, betaine, dimethylglycine, SAM, and SAH may provide additional mechanistic information. Such multiparametric assessment remains a research approach and is not yet a validated clinical algorithm.

Longitudinal assessment may provide additional insight into the relative contributions of production, metabolism, and clearance. Temporal changes, particularly in response to dietary interventions, vitamin supplementation, antibiotic exposure, or variations in renal function, may facilitate the identification of the predominant underlying mechanism and overcome the limitations of static measurements [4,6,57,58].

## 5. Analytical and Pre-Analytical Considerations

**Figure 3:**
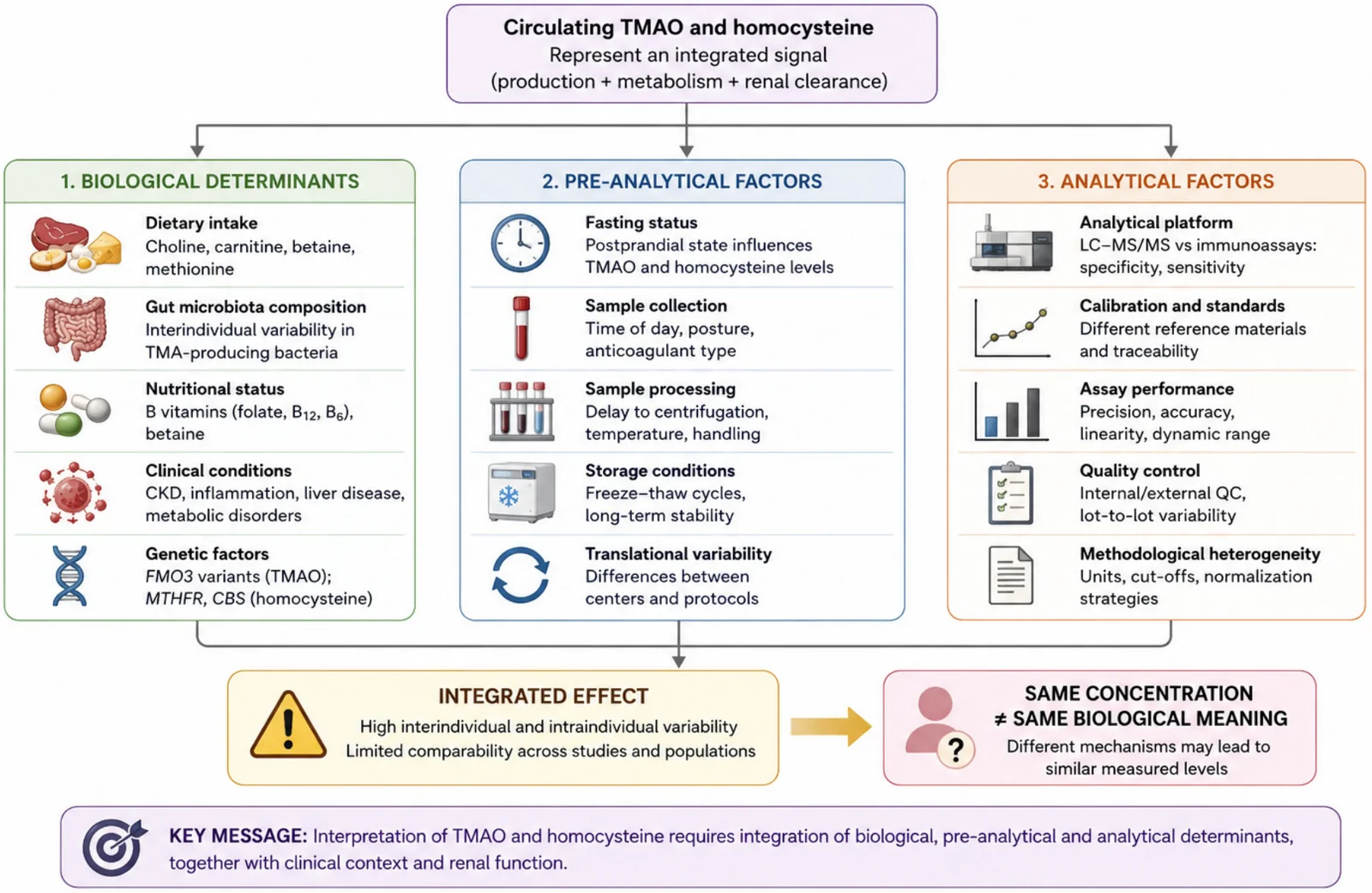
Sources of variability in TMAO and homocysteine measurement and interpretation. Validated analytical methods allow reliable quantification, but circulating concentrations remain influenced by renal function, diet, gut microbiota, nutritional status, sample handling, and analytical methodology. These factors should be standardized or reported when the biomarkers are used in clinical research.

Accurate measurement and interpretation of TMAO and homocysteine require careful consideration of analytical and pre-analytical variables, which represent important sources of variability [13]. Validated methods can quantify both analytes reliably; the principal challenge is the comparability and biological interpretation of results obtained under different sampling and clinical conditions (Figure 3).

### 5.1. Pre-Analytical Considerations

Pre-analytical variables are of particular significance for the measurement of homocysteine. Whole blood homocysteine concentrations increase progressively after sampling because of continued release from erythrocytes, making rapid sample processing essential. It has been demonstrated that delayed centrifugation may result in the overestimation of plasma concentrations, with increases reported ranging from 10 to 15% per hour at room temperature. It is therefore recommended that prompt plasma separation or the use of stabilising collection tubes is employed [59,60].

For TMAO, recent dietary exposure is a major pre-analytical and biological determinant. Fish may contain preformed TMAO, whereas meals containing choline or carnitine may alter postprandial TMAO depending on microbial metabolic capacity. Standardized fasting sampling is therefore reasonable when basal concentrations are compared, although fasting cannot remove the effects of habitual diet or microbiota composition. Recent fish intake, supplements containing choline, betaine, carnitine or B vitamins, antibiotic use, and relevant medications should be recorded.

Both biomarkers are also influenced by longer-term nutritional and metabolic factors. Vitamin B12, folate, and vitamin B6 status strongly affect homocysteine metabolism [33], whereas dietary patterns and gut microbiota composition significantly modulate TMAO production [6,61,62]. These variables should therefore be considered during interpretation process, especially in the context of heterogeneous clinical populations.

### 5.2. Analytical Considerations

Liquid chromatography–tandem mass spectrometry (LC–MS/MS), particularly with stable-isotope internal standards, enables specific and sensitive quantification of TMAO and related metabolites. Accordingly, TMAO measurement itself should not be presented as a major analytical challenge [13,63].

Interlaboratory differences may nevertheless arise from calibration, internal standards, sample preparation, chromatographic conditions, matrix effects, and the lack of universally harmonized reference procedures and clinical decision limits [7,62].

Homocysteine can be measured using immunochemical and chromatographic techniques, including HPLC and LC–MS/MS. Immunoassays provide high throughput, whereas chromatographic methods offer greater analytical specificity. The analytical performance of total homocysteine is comparatively well established, although method-dependent bias and pre-analytical error remain relevant [59,60,64].

### 5.3. Interpretative Implications

The interpretation of biomarkers is complicated by analytical and pre-analytical variability. For TMAO, biological variability related to diet, gut microbiota, and renal function may limit its reliability as a standalone biomarker even when analytical measurement is robust [13]. For homocysteine, inadequate sample handling remains an important source of error and may produce clinically misleading elevations [59].

Consequently, the interpretation of TMAO and homocysteine concentrations should always be contextualised within relevant clinical and laboratory information, including fasting status, renal function, nutritional parameters, and analytical methodology. Standardization can reduce variability but cannot make either metabolite a kidney-specific biomarker.

## 6. Nutritional Interface and Shared Downstream Mechanisms

TMAO-generating pathways and one-carbon metabolism are distinct. Their principal biochemical interface is the availability of choline and betaine. Choline may be used by gut microorganisms for TMA formation or oxidized by the host to betaine, whereas betaine supports BHMT-dependent homocysteine remethylation. This substrate interface does not demonstrate a direct competitive axis. Current human evidence is insufficient to establish that diversion of dietary choline toward microbial TMA production depletes the systemic choline–betaine pool, impairs BHMT activity, and causes hyperhomocysteinemia [27,28,61].

Within one-carbon metabolism, the SAM/SAH ratio is an important determinant of cellular methylation capacity because SAH acts as a potent product inhibitor of most methyltransferases. Hyperhomocysteinemic conditions may promote intracellular SAH accumulation and reduce the SAM/SAH ratio, thereby contributing to global and gene-specific changes in DNA, RNA, protein, and histone methylation [45,65]. Recent experimental evidence suggests that TMAO may also inhibit S-adenosylhomocysteine hydrolase, promote SAH accumulation, and alter histone methylation; however, these findings derive from cultured cells and mouse models and have not yet been demonstrated in humans [66].

Recent studies have demonstrated altered methylation of genes involved in endothelial nitric oxide synthase (eNOS) signaling, extracellular matrix remodeling, and inflammatory responses under hyperhomocysteinemic conditions [65,67]. These epigenetic changes may contribute to endothelial dysfunction and progressive renal injury.

Emerging evidence further suggests that disturbances in choline-betaine metabolism may influence methylation capacity and thereby indirectly connect TMAO-related pathways with epigenetic regulation. However, direct evidence linking circulating TMAO concentrations with specific epigenetic signatures in humans remains limited [66].

Concurrently, both TMAO and homocysteine have been independently associated with mechanisms implicated in cardiovascular and renal disease, including oxidative stress, inflammation, endothelial dysfunction, and fibrosis [13,41,68]. These partially overlapping downstream responses provide a potential pathophysiological convergence, but they do not demonstrate synergy, direct interaction, or a common biosynthetic pathway.

Renal dysfunction may amplify these responses through metabolite retention, changes in the intestinal environment, and the broader inflammatory and metabolic disturbances of CKD. However, the relative contributions of each metabolite and of renal dysfunction itself remain difficult to separate. Studies simultaneously evaluating TMAO, homocysteine, one-carbon intermediates, diet, and kidney function are needed before mechanistic interdependence can be established.

The majority of available data are derived from experimental or associative studies, while clinical investigations simultaneously evaluating TMAO, homocysteine, methylation intermediates, and kidney function are still scarce.

Recent experimental evidence indicates that both TMAO and homocysteine may influence cellular stress responses through partially overlapping molecular mechanisms. TMAO has been associated with activation of endoplasmic reticulum (ER) stress pathways involving Protein kinase R (PKR)-like endoplasmic reticulum kinase (PERK), Inositol-Requiring Enzyme 1 alpha (IRE1α), and Activating Transcription Factor 6 (ATF6) signaling. Sustained ER stress may subsequently promote inflammation, apoptosis, and fibrotic remodeling [69,70,71].

Homocysteine has similarly been shown to induce ER stress and mitochondrial dysfunction through excessive ROS production, altered calcium homeostasis, and impairment of mitochondrial respiratory chain activity. These mechanisms converge on apoptotic signaling pathways involving Bax/Bcl-2 imbalance, cytochrome c release, and caspase activation [72,73].

**Figure 4:**
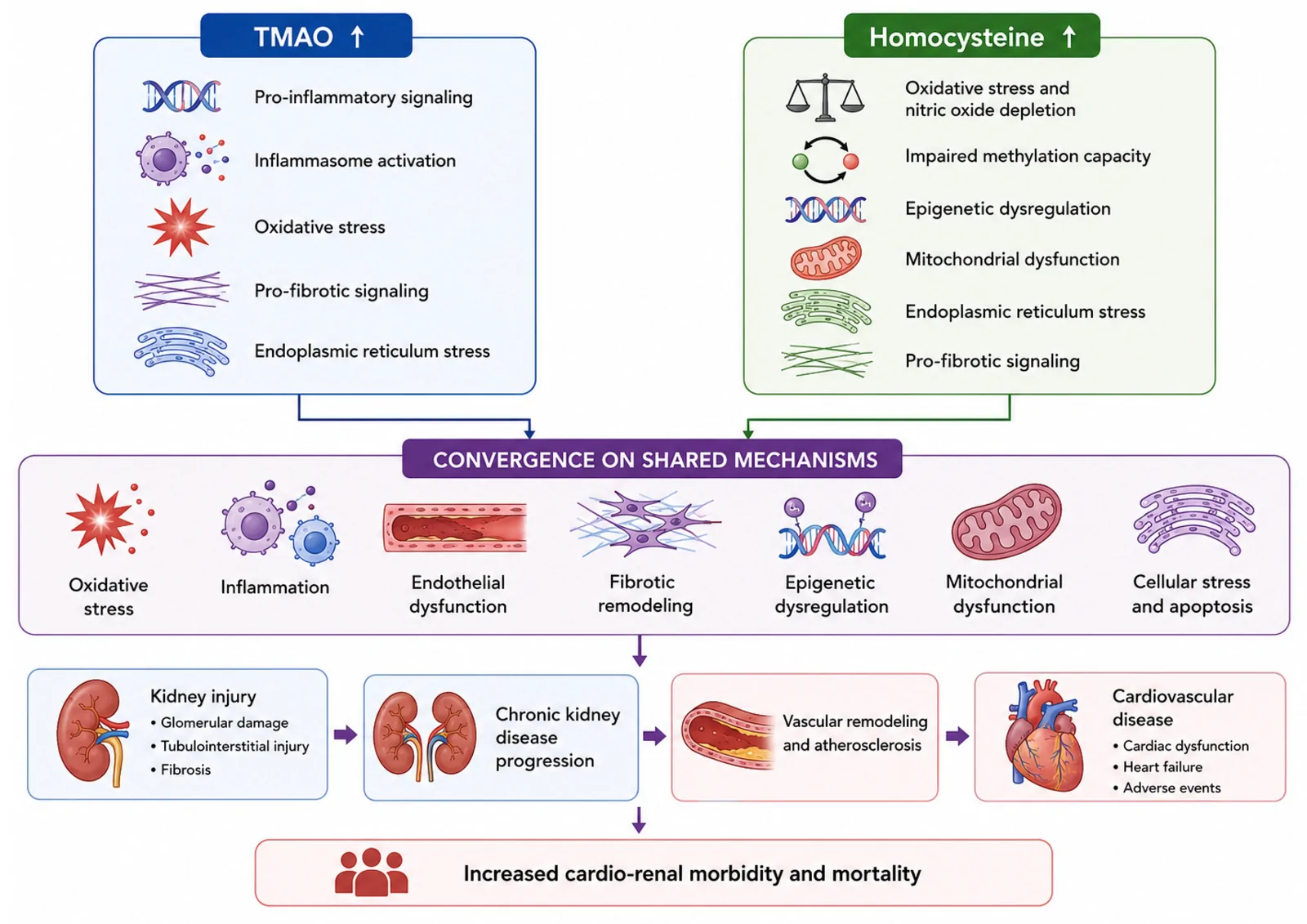
Partially overlapping downstream mechanisms associated with TMAO and hyperhomocysteinemia. Despite their distinct metabolic origins, experimental evidence links both metabolites to oxidative stress, inflammation, endothelial dysfunction, cellular stress, apoptosis, and fibrotic remodeling. These shared associations represent potential pathophysiological convergence but do not demonstrate a common biosynthetic pathway or equivalent causal contribution to cardiorenal disease.

The convergence of oxidative stress, mitochondrial dysfunction, ER stress, and inflammatory signaling may therefore represent a common molecular framework linking elevated TMAO and homocysteine concentrations to cardio-renal pathology (Figure 4).

## 7. Nutritional Implications and the Therapeutic Paradox in CKD

Dietary modulation of TMAO-generating pathways in CKD requires caution. Choline is an essential nutrient involved in membrane phospholipids, neurotransmission, lipid transport, and methyl-group metabolism, while betaine supports BHMT-dependent homocysteine remethylation [27,62]. Current evidence does not support restricting choline or betaine solely to reduce circulating TMAO, particularly in patients at risk of malnutrition.

The effects of dietary interventions depend on food source, overall dietary pattern, gut microbiota composition, and kidney function [6,37,61]. Nutritional management should therefore prioritize established CKD recommendations and adequate nutritional status. Controlled studies are needed to determine whether dietary or microbiota-directed strategies can reduce TMAO without adversely affecting essential nutrient pathways.

## 8. Clinical Implications and Limitations

The clinical interpretation of TMAO and homocysteine requires careful contextualization within a broader metabolic and renal framework. Despite the consistent association of both biomarkers with cardiovascular and CKD risk, their clinical utility remains limited by the multifactorial nature of their circulating concentrations [4,13].

A significant restriction inherent to single-point measurements is their inability to discern whether elevated concentrations are predominantly attributable to augmented production, modified metabolism, diminished renal clearance, or a confluence of these processes. This issue assumes particular relevance in cases of CKD, where impaired renal elimination has been demonstrated to amplify circulating concentrations. The underlying metabolites of the affected subjects may still participate in, or reflect, pathogenic pathways associated with cardio-renal risk. The primary interpretative challenge is therefore not whether elevated TMAO or homocysteine concentrations are clinically relevant, but whether they indicate active metabolic dysregulation, renal retention, or both. Renal function should consequently be considered when evaluating these biomarkers. Associations with clinical outcomes should be reported before and after adjustment for eGFR and established cardiovascular or renal risk factors. Neither TMAO nor homocysteine is currently an established replacement for creatinine, cystatin C, albuminuria, or eGFR in the diagnosis of kidney disease. Renal dysfunction therefore reduces the mechanistic specificity of both biomarkers, although it does not necessarily eliminate an observed prognostic association.

The presence of additional sources of variability serves to further complicate the interpretation process. TMAO concentrations are significantly influenced by dietary intake and the composition of gut microbiota, whereas homocysteine levels are substantially affected by vitamin B12, folate, and vitamin B6 status [6,33,36]. Analytical and pre-analytical factors have also been demonstrated to contribute substantially to variability, including methodological heterogeneity for TMAO measurement and the well-established instability of homocysteine in whole blood samples [13,59].

Another significant limitation pertains to the concept of causality. Despite the fact that elevated TMAO and homocysteine concentrations have been repeatedly associated with adverse cardiovascular and renal outcomes, it remains uncertain to what extent these metabolites represent direct mediators of disease rather than markers of broader metabolic dysfunction or impaired renal clearance and observational associations do not demonstrate that lowering either metabolite will improve clinical outcomes [50,55,56]. This distinction is clinically relevant because biomarkers reflecting systemic metabolic stress may still retain prognostic value even in the absence of direct pathogenicity.

The isolated measurement of TMAO or homocysteine is unlikely to provide sufficient biological or clinical insight. There is also insufficient evidence to recommend the TMAO/homocysteine ratio for clinical use; a ratio may combine rather than resolve the determinants and variability of its two components. Multiparametric assessment may support mechanistic research but requires validation before routine application.

The clinical evidence should therefore be interpreted according to a hierarchy. Experimental studies establish biological plausibility; cross-sectional studies describe associations with CKD severity; longitudinal studies can evaluate temporal relationships and prognosis; and intervention studies are required to determine whether modifying the biomarker or its pathway improves outcomes. For TMAO, prospective studies have reported associations with incident CKD, kidney function decline, and mortality, including associations persisting after multivariable adjustment, but targeted outcome trials are lacking [50,51]. For homocysteine, the lack of clinical benefit despite substantial lowering with folic acid and B-vitamin supplementation in randomized trials involving patients with advanced CKD or end-stage kidney disease indicates that lowering the circulating concentration does not necessarily translate into improved clinical outcomes [55,56].

Accordingly, the potential prognostic value of these metabolites must be distinguished from diagnostic specificity, causal relevance, and therapeutic utility. A biomarker may predict an outcome without identifying the mechanism responsible for that risk, and statistical independence from eGFR does not automatically establish clinically meaningful incremental value. Future studies should compare models with and without TMAO or homocysteine using discrimination, calibration, reclassification, and decision-analytic measures, followed by external validation. Until such evidence is available, their principal role remains investigational and complementary to established renal and cardiovascular assessment.

## 9. Future Perspectives

Future research should focus on clarifying the dynamic interactions between TMAO-related pathways, one-carbon metabolism, gut microbiota activity, and renal function. Longitudinal studies using standardized sampling and validated assays should characterize renal function, diet, nutritional status, medication, supplementation, and microbiota-modifying exposures.

Multiparametric approaches incorporating metabolites such as betaine, dimethylglycine, SAM, and SAH may provide a more integrated representation of metabolic and cardio-renal homeostasis than isolated biomarker measurements alone. Such studies should test whether coordinated changes represent true mechanistic interaction or parallel responses to shared determinants.

Further work is also needed to quantify glomerular and tubular contributions to human TMAO handling, determine whether hepatic FMO3 activity is altered in human CKD, and clarify the metabolic basis of CKD-associated hyperhomocysteinemia. Analytical harmonization and optimization of pre-analytical protocols remain important for comparing results across studies.

Multi-omics approaches integrating metabolomics, epigenomics, transcriptomics, and microbiome profiling may provide a deeper insight into the relationships among TMAO, homocysteine, and renal dysfunction.

Ultimately, intervention studies must determine whether modifying either pathway improves patient-centered outcomes rather than merely changing biomarker concentrations.

## 10. Conclusions

The comparison of TMAO and homocysteine reveals that their relevance to kidney disease lies less in a shared metabolic origin than in the way renal dysfunction changes the meaning of their circulating concentrations. For TMAO, the strong dependence on glomerular filtration, the marked accumulation in advanced CKD, and the reduction after restoration of kidney function indicate that renal clearance is a major determinant of exposure. For homocysteine, the relationship with CKD is metabolically more complex and cannot be explained by urinary retention alone.

The evidence also supports a distinction between accumulation, biological activity, and causality. Experimental studies provide plausible mechanisms through which both metabolites may contribute to oxidative, inflammatory, endothelial, and fibrotic injury, whereas human studies consistently demonstrate associations with renal dysfunction and adverse outcomes. However, residual confounding and reverse causation remain difficult to exclude. This limitation is particularly evident for homocysteine, for which substantial lowering with folic acid and B vitamins has not translated into improved outcomes in advanced CKD. For TMAO, prospective associations with incident CKD and kidney function decline warrant further investigation, but targeted intervention trials are still lacking. Accordingly, shared downstream effects should be regarded as a pathophysiological hypothesis supported by experimental and associative evidence rather than proof of a unified causal axis.

These considerations define both the potential and the current limits of clinical application. Neither TMAO nor homocysteine can presently replace established assessment based on eGFR, albuminuria, creatinine, cystatin C, and conventional cardiovascular risk factors. Their most informative use is likely to be contextual and hypothesis-driven: TMAO may help characterize the gut–liver–kidney contribution to metabolic exposure, whereas homocysteine may reflect disturbances in one-carbon metabolism, vitamin status, and the uremic metabolic environment. Future studies should determine whether either analyte provides reproducible incremental prognostic information and, more importantly, whether pathway-directed interventions improve renal or cardiovascular outcomes. Until then, they should be considered complementary research biomarkers whose interpretation depends on renal function, nutritional status, and the broader metabolic setting.

## Data Availability

No new data were created or analyzed in this study. Data sharing is not applicable to this article.

## Abbreviations

The following abbreviations are used in this manuscript:

TMAO	Trimethylamine N-oxide
CKD	Chronic kidney disease
BHMT	Betaine homocysteine S methyltransferase
eGFR	estimated glomerular filtration rate
NF-κB	Nuclear factor-kappa B
NLRP3	NOD-like receptor protein 3
TGF-β	Transforming growth factor-β
ROS	Reactive oxygen species
SAM	S-adenosylmethionine
SAH	S-adenosylhomocysteine
TMA	Trimethylamine
FMO3	Flavin-containing monooxygenase 3
IL-1β	Interleukin-1 beta
IL-6	Interleukin-6
TNF-α	Tumor necrosis factor-alpha
IL-18	Interleukin-18
NO	Nitric oxide
LC–MS	Liquid chromatography–tandem mass spectrometry
HPLC	High-performance liquid chromatography
DMG	Dimethylglycine
hHCy	hyperhomocysteinemia
eNOS	Endothelial nitric oxide synthase
ER	Endoplasmic reticulum
PERK	Protein kinase R (PKR)-like endoplasmic reticulum kinase
IRE1α	Inositol-Requiring Enzyme 1 alpha
ATF6	Activating Transcription Factor 6
